# Marital Status and Risk of Dementia: Does Race Matter?

**DOI:** 10.1093/geroni/igaa057.2683

**Published:** 2020-12-16

**Authors:** Zhenmei Zhang, Hui Liu, Seung-won Choi

**Affiliations:** 1 Michigan State University, Okemos, Michigan, United States; 2 Michigan State University, EAST LANSING, Michigan, United States; 3 Texas Tech University, Lubbock, Texas, United States

## Abstract

Previous research has shown that unmarried individuals (i.e., divorced, widowed, and never married) had a higher risk of dementia than their married counterparts. However, few studies examined whether the link between marital status and dementia varies by race. To fill the gap, we used data from the Health and Retirement Longitudinal Study (2000-2014) and analyzed 15,379 respondents (13,278 non-Hispanic whites and 2,101 non-Hispanic blacks) ages 50 and older in 2000 who had no dementia. Discrete-time event history models were estimated. Our preliminary analysis showed that marital status was significantly associated with the odds of dementia for both whites and blacks. Furthermore, the associations between unmarried status (i.e., cohabiting, widowed, and never married) and dementia were stronger among blacks than whites. The effect of divorce on odds of dementia did not differ by race. The results were robust after controlling for socioeconomic status, health and lifestyle factors, and social engagements. Part of a symposium sponsored by the Alzheimer’s Disease Research Interest Group.

